# Coupled molecular dynamics mediate long- and short-range epistasis between mutations that affect stability and aggregation kinetics

**DOI:** 10.1073/pnas.1810324115

**Published:** 2018-11-07

**Authors:** Haoran Yu, Paul A. Dalby

**Affiliations:** ^a^Department of Biochemical Engineering, University College London, London WC1H 0AH, United Kingdom

**Keywords:** dynamics, epistasis, stability, protein engineering, transketolase

## Abstract

Multiple mutations are typically required to significantly improve protein stability or aggregation kinetics. However, when several substitutions are made in a single protein, the mutations can potentially interact in a nonadditive manner, resulting in epistatic effects, which can hamper protein-engineering strategies to improve thermostability or aggregation kinetics. Here, we have examined the role of protein dynamics in mediating epistasis between pairs of mutations. With *Escherichia coli* transketolase (TK) as a model, we explored the epistatic interactions between two single variants H192P and A282P, and also between the double-mutant H192P/A282P and two single variants, I365L or G506A. Epistasis was determined for several measures of protein stability, including the following: the free-energy barrier to kinetic inactivation, ∆∆*G*^‡^; thermal transition midpoint temperatures, *T*_m_; and aggregation onset temperatures, *T*_agg_. Nonadditive epistasis was observed between neighboring mutations as expected, but also for distant mutations located in the surface and core regions of different domains. Surprisingly, the epistatic behaviors for each measure of stability were often different for any given pairwise recombination, highlighting that kinetic and thermodynamic stabilities do not always depend on the same structural features. Molecular-dynamics simulations and a pairwise cross-correlation analysis revealed that mutations influence the dynamics of their local environment, but also in some cases the dynamics of regions distant in the structure. This effect was found to mediate epistatic interactions between distant mutations and could therefore be exploited in future protein-engineering strategies.

## Significance

Incomplete understanding of the mechanisms of epistasis between two or more substitutions in a protein can hamper protein-engineering strategies. With *Escherichia coli* transketolase as a model, we explore the epistatic interactions between a set of stabilizing mutations from across two different domains within the protein structure. Surprisingly, not all pairwise effects between distant mutations from the surface and core regions of different domains were additive. Additionally, the epistatic behaviors observed were dependent on the type of stability measured. We found single mutations that altered local protein dynamics, which then induced correlated changes in the dynamics of a second domain of the same monomer. This mechanism mediated epistasis between distant mutations.

Proteins and enzymes are increasingly used as therapeutics, as diagnostics, and for industrial biocatalysis. These applications often require function at elevated temperatures or after long-term storage, and so the development of efficient strategies to enhance their stability remains a major goal in protein engineering. High thermal stability is also strongly correlated with expression yield, in vitro half-life, and in vivo serum survival time (1–3). Additionally, thermostable proteins tolerate more mutations than mesophilic ones, which makes them a better starting point in protein engineering (3, 4).

Directed evolution is a powerful strategy for engineering protein stability through the accumulation of beneficial substitutions. The desired property is obtained by screening or selection of a library of randomly mutated variants. However, it is often necessary to screen large numbers of mutants in several rounds of evolution to obtain a desired level of change (5). Where there is no high-throughput screen for the target property, such as aggregation kinetics of purified enzymes, then random mutagenesis approaches become inaccessible. By contrast, smart-library design and semirational site-directed mutagenesis has gained popularity due to improved efficiency (6) and improvements in the prediction accuracy with which computational or rational design strategies can propose stabilizing mutations. For example, reconstructed ancestor or consensus sequences from multiple protein sequence alignments can propose mutations based on the hypothesis that they are more thermostable than extant homologs (7, 8). Statistical analysis of protein secondary-structure sequences also found that proline prefers to be at the second position of β-turns (9). The strategy of inserting proline mutations is also well known to stabilize many enzymes (10, 11). Meanwhile, structural information has been critical for computational design methods. Many algorithms apply geometrical or energy constraints when analyzing 3D structures, to optimize the surface charge of proteins, or introduce disulfide bonds that increase protein stability (12, 13). Recently, several computational protein design algorithms have been developed to predict the impact of mutations upon stability to global unfolding, including Rosetta (14), FoldX (15), and SDM (16).

While site-directed mutagenesis is widely used to engineer protein stability, single-point mutations usually contribute relatively little, and multiple mutations are typically required to stabilize large proteins (17). Many mutations contribute independently to fitness, and their collective contributions (Δ_AB_ for A and B) are found to be mathematically additive, where Δ_AB_ = Δ_A_ + Δ_B_. The contributions of some are affected by mutations made at other sites in the protein, in a phenomenon known as intragenic epistasis (18). Therefore, when mutations that contribute positively on their own (Δ_A_ > 0) are combined into a single protein, two or more mutations often interact in a nonadditive manner. This epistatic behavior can be measured via its effect on various protein properties and can have either positive epistasis, where Δ_AB_ = Δ_A_ + Δ_B_ + X; negative epistasis (partially additive), where (Δ_A_ and Δ_B_) < Δ_AB_ < Δ_A_ + Δ_B_; negative sign epistasis, where Δ_AB_ < (Δ_A_ or Δ_B_); or reciprocal sign epistasis, where Δ_AB_ < 0 (19–21). Additive effects are most likely when the structural regions influenced by each mutation do not substantially overlap (22). It is well known that epistasis is most likely for mutated residues that are in direct contact with each other (23, 24). However, epistasis has also been observed between mutations of structurally distant residues, with their effects proposed to be mediated through a network of interactions (25). How such networks mediate epistasis between distant mutations remains poorly understood and thus hampers the development of more effective rational or semirational protein-engineering strategies (26).

Dynamics potentially mediate long-range communication in proteins (27). Several studies have investigated the impact of point mutations upon protein dynamics using NMR (28) or computational algorithms (29, 30), and found that changes in the dynamics due to single point mutations could be frequent, significant, and long-ranged. Most studies have focused on the impact of long-range dynamics on allostery, ligand binding, and the effect of mutations distant from sites normally associated with function (31). However, little is known about the role of dynamics in long-range epistasis between mutations, or their impact on conformational stability, and even less for aggregation kinetics.

With *Escherichia coli* transketolase (TK) as a model, we investigated how the combination of stabilizing mutations influenced various measures of protein stability, including thermal transition midpoint (*T*_m_), aggregation onset temperature (*T*_agg_), rates of irreversible thermal inactivation at elevated temperature, and the fraction unfolded at that temperature (*f*_T_). For each, we explored the additivity for pairs of mutations, compared their structural locations, and investigated their impact on protein flexibility to determine the role of dynamics in epistasis. TK, a thiamine diphosphate-dependent (ThDP) enzyme, catalyses the reversible transfer of a C2-ketol unit from d-xylulose-5-phosphate to either d-ribose-5-phosphate or d-erythrose-4-phosphate in living cells (32, 33). TK is a homodimer of two 70- to 74-kDa monomers, each composed of a pyrophosphate (PP)-binding domain, pyrimidine (Pyr)-binding domain, and a C-terminal domain. A Mg^2+^ or Ca^2+^ ion, and ThDP cofactor binds into each active site formed at the two identical interfaces between the PP and Pyr domains of opposite subunits. TK has considerable industrial biocatalytic potential for the stereospecific synthesis of carbon–carbon bonds in complex carbohydrates and other high-value compounds (34, 35). Use of β-hydroxypyruvate (HPA) as the ketol donor renders the donor-half reaction irreversible, thus increasing the atom efficiency of the reaction favorably for industrial syntheses. *E. coli* TK converts HPA with a rate of 60 U/mg, significantly higher than the 2 and 9 U/mg reported for its orthologs from spinach and yeast (36).

Directed evolution has expanded the ability of *E. coli* TK to accept a wide range of nonnatural substrates (37–40). However, as a mesophilic enzyme, *E. coli* TK suffers from poor stability at elevated temperatures and extremes of pH, which has hampered its wide application in industrial processes (41). Recently, we constructed mutants focused at different regions of *E. coli* TK to increase its thermostability. First, by mutating residues in the flexible cofactor-binding loops toward those found in *Thermus thermophilus* at equivalent positions, the H192P mutation was found to double the half-life at 60 °C (42, 43). Using the rigidifying flexible sites strategy (44), 49 single mutations were individually targeted to flexible loops on the surface, which led to several more stable variants including D143K, I189H, and A282P. Combining H192P with A282P extended the half-life at 60 °C to triple that of WT (42). In a separate study, consensus mutations were targeted to protein hydrophobic core regions, and six single mutants including I365L, G506A, and V228I showed significantly improved thermostability compared with WT (45).

Here, we created a set of variants along different evolutionary pathways from WT to H192P/A282P/I365L/G506A, based on four individually thermostabilizing single mutations identified previously. H192P and A282P were located 33 Å apart on the surface of the PP-binding domain, whereas I365L and G506A were located 12 Å apart in the hydrophobic core of the Pyr-binding domain, and at least 25 Å from H192P or A282P. The PP and Pyr domains from opposite chains interact strongly with each other in the homodimer, and so mutations in each domain might be expected to influence those in the other through either an interchain or intrachain mechanism. We generated four new variants, I365L/G506A, H192P/A282P/I365L, H192P/A282P/G506A, and H192P/A282P/I365L/G506A, and then investigated the epistatic interactions among the four mutation sites by analyzing the kinetics and free energy of thermal inactivation, *T*_m_, *T*_agg_, and *f*_T_, for all variants. Molecular-dynamics (MD) simulations were analyzed using a dynamics cross-correlation matrix to reveal the role of dynamics in mediating the observed epistasis between mutations.

## Results and Discussion

### Design of Combined Mutations.

**Fig. 1.**
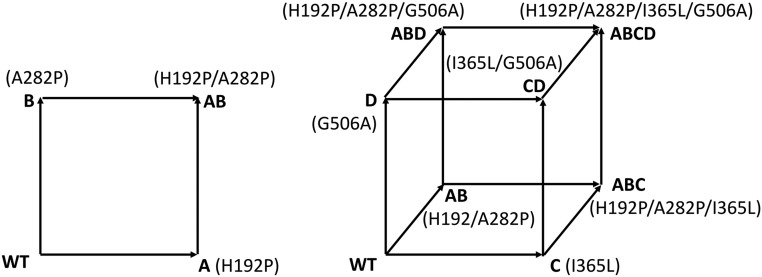
Graphical representation of sequence space from WT to the thermostable mutant H192P/A282P/I365L/G506A (ABCD), where each vertex of the cube represents a variant and each edge represents a single mutational step. Each variant was represented by letter(s): A—H192P, B—A282P, C—I365L, D—G506A, AB—H192P/A282P, CD—I365L/G506A, ABC—H192P/A282P/I365L, ABD—H192P/A282P/G506A, and ABCD—H192P/A282P/I365L/G506A.

Variants H192P, A282P, H192P/A282P, I365L, and G506A were obtained previously from two different strategies in which mutations were targeted to surface loop, and hydrophobic core regions of *E. coli* TK, respectively. Here, we created variants along a set of evolutionary pathways but divided into two steps. The first step combined H192P (A) and A282P (B) into a double mutant, H192P/A282P (AB), as previously, to explore the epistasis between A and B. The second step combined three variants I365L (C), G506A (D), and H192P/A282P (AB) to generate one double, I365L/G506A (CD); two triple, H192P/A282P/I365L (ABC) and H192P/A282P/G506A (ABD); and a quadruple mutant, H192P/A282P/I365L/G506A (ABCD). This enabled us to measure the additive and nonadditive effects from across the eight (=2^3^) possible variants, combined in six (=3!) possible forward pathways from WT: P1, C-CD-ABCD; P2, C-ABC-ABCD; P3, D-CD-ABCD; P4, D-ABD-ABCD; P5, AB-ABC-ABCD; P6, AB-ABD-ABCD (Fig. 1).

### Comparison of Kinetic and Thermodynamic Stability.

**Fig. 2.**
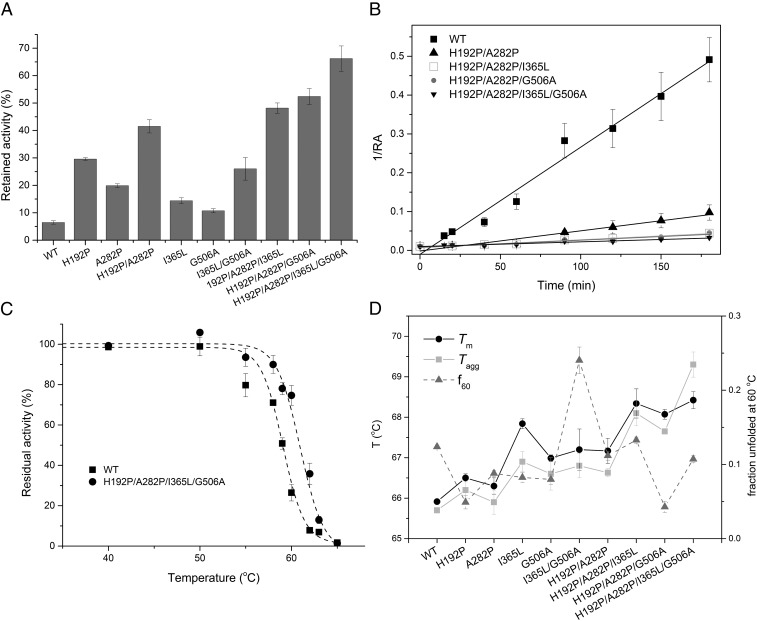
Thermal stability of TK and its variants. (*A*) Activity retained after heating at 60 °C for 1 h. Data were averaged from triplicate experiments and the SD is shown in parentheses. (*B*) Second-order degradation kinetics of TK variants at 60 °C. RA represents residual activity. WT, *y* = 0.00275*x* − 0.0085, *R*^2^ = 0.98; H192P/A282P, *y* = 0.00052*x* − 0.0016, *R*^2^ = 0.95; H192P/A282P/I365L, *y* = 0.000197*x* + 0.0076, *R*^2^ = 0.98; H192P/A282P/G506A, *y* = 0.000187*x* + 0.0075, *R*^2^ = 0.94; H192P/A282P/I365L/G506A, *y* = 0.000121*x* + 0.0099, *R*^2^ = 0.95. (*C*) Thermal-induced inactivation of WT and a quadruple variant. Enzymes in 100 µL of solution (2.4 mM TPP, 9 mM MgCl_2_, and 50 mM Tris⋅HCl, pH 7.0) were incubated at different temperature from 22 to 65 °C for 15 min and assayed for residual activity at 22 °C. The activity incubated at 22 °C was considered to be 100%. (*D*) *T*_m_, *T*_agg_, and fraction unfolded at 60 °C (*f*_60_) for WT and variant TKs.

For industrial enzymes, kinetic stability is critical for retaining activity during the time course of the bioconversion at a given operating temperature. The kinetic stability of TK at elevated temperatures relates to the rate of inactivation due to irreversible aggregation, promoted by increased partial unfolding of the native protein upon heating (41). The kinetic stability of TK variants was determined from the activity retained after incubation at 60 °C for 1 h. The combined variants all retained higher activities than the WT and respective single-mutant parents. The quadruple mutant, H192P/A282P/I365L/G506A, retained 66.2% activity after incubation at 60 °C for 1 h, representing a 10.2-fold improvement over WT (Fig. 2*A*). To probe the kinetic stabilities of the TK combined variants in more detail, we measured their half-lives, *t*_1/2_, for loss of enzyme activity at 60 °C, by incubating them at 60 °C for different periods of time (Fig. 2*B* and *SI Appendix*, Fig. S1). Whereas, in previous work, inactivation profiles were fit with less accuracy to single-exponential decays (42, 43), giving longer estimates of half-lives, the current analysis revealed better fits to a second-order reaction equation. The half-lives for WT, H192P, A282P, and H192P/A282P were now determined as 4, 15.2, 7.7, and 19.3 min, respectively (Table 1). All three newly combined variants shown in Fig. 2*B* deactivated more slowly than both WT and H192P/A282P, indicating an increased resistance to high temperature. The two triple-mutant variants, H192P/A282P/I365L and H192P/A282P/G506A, had similar half-lives, 50.6 and 53.2 min, respectively (Table 1). The quadruple variant, H192P/A282P/I365L/G506A, had the highest half-life of 82.5 min, representing a 21-fold improvement over that of WT (4 min). Enzyme kinetic studies revealed that H192P/A282P/I365L, H192P/A282P/G506A, and H192P/A282P/I365L/G506A did not appear to impact significantly on the kinetic parameters *k*_cat_ and *K*_m_, indicating their improved potential to be used for biocatalysis (*SI Appendix*, Table S1).

**Table 1.** Thermal stabilities of WT and mutant TKs

TK	*T*_agg_, °C	*T*_m_, °C	*∆S*_vh_, kcal⋅mol^−1^⋅K^−1^	*f*_60_	*k*_d_ × 10^3^, %^−1^⋅min^−1^	*t*_1/2_, min	*T*_50_^15^, °C
WT	65.7 (0.07)*	65.7 (0.04)	0.23 (0.012)	0.12 (0.01)	2.80 (0.04)	4.0 (0.3)	58.5 (0.4)
H192P	66.2 (0.2)	66.5 (0.1)	0.32 (0.03)	0.049 (0.009)	0.66 (0.05)	15.2 (1.2)	ND
A282P	65.9 (0.3)	66.3 (0.2)	0.25 (0.01)	0.088 (0.004)	1.29 (0.03)	7.7 (0.2)	ND
I365L	66.9 (0.2)	67.1 (0.1)	0.25 (0.04)	0.082 (0.008)	ND	ND	ND
G506A	66.6 (0.4)	66.7 (0.08)	0.22 (0.002)	0.08 (0.009)	ND	ND	ND
I365L/G506A	66.8 (0.3)	67.2 (0.5)	0.078 (0.01)	0.24 (0.03)	ND	ND	ND
H192P/A282P	66.6 (0.1)	67.4 (0.3)	0.19 (0.013)	0.11 (0.01)	0.54 (0.06)	19.3 (1.3)	ND
H192P/A282P/I365L	68.1 (0.3)	68.6 (0.4)	0.13 (0.01)	0.13 (0.02)	0.20 (0.02)	50.6 (3.3)	ND
H192P/A282P/G506A	67.6 (0.05)	68.1 (0.3)	0.19 (0.003)	0.043 (0.007)	0.19 (0.02)	53.2 (3.5)	ND
H192P/A282P/I365L/G506A	69.3 (0.5)	68.9 (0.4)	0.17 (0.007)	0.11 (0.02)	0.12 (0.01)	82.5 (5.7)	61.0 (0.5)

ND, not determined.

* SEMs were calculated from triplicate experiments and listed in parentheses.

The quadruple-variant and WT TK were also incubated at different temperatures from 22 to 65 °C for 15 min, and the retained activities measured after cooling to 22 °C were calculated relative to those incubated throughout at 22 °C. No significant differences in residual activity were observed with incubations below 50 °C (Fig. 2*C*). However, incubation at 57 °C reduced the activity of WT 71.7%, whereas the quadruple variant retained 90.0% of the original activity. The temperature required to reduce the initial enzyme activity by 50% within 15 min (*T*_50_^15^), for WT was around 58.5 °C, which was 2.5 °C lower than that of the quadruple variant (61.0 °C) (Table 1). At 50 °C, the retained activity of the quadruple variant increased by 5% compared with lower temperatures. As we have observed previously, heat treatment at 50 °C improved the activity of WT *E. coli* TK by 50% after 0.5 h, and by 100% after 1 h (41), while heat treatment at 55 °C increased the activity of H192P by 2.5-fold after 1 h (43). The more limited activity improvement after incubation at 50 °C in the present work was due to the shorter incubation time of only 15 min.

We also investigated the thermal transition midpoint temperatures, *T*_m_, a measure of thermodynamic conformational stability obtained from intrinsic fluorescence measurements, for all variants. Their aggregation onset temperatures, *T*_agg_, were simultaneously determined from static light scattering measurements (Fig. 2*D*). All variants had increased *T*_m_ and *T*_agg_ compared with those of WT (Fig. 2*D*). The three variants H192P, A282P, and H192P/A282P had *T*_m_ values 0.8, 0.6, and 1.7 °C higher, respectively, than that of WT. The quadruple mutant had the highest thermodynamic stability, with *T*_m_ and *T*_agg_ values 3.2 and 3.6 °C higher, respectively, than those of WT. *T*_agg_ values were ∼0.2–0.5 °C lower than *T*_m_ in all cases, except for the quadruple mutant, for which *T*_agg_ was 1 °C higher than *T*_m_. This close link indicated that, on the timescale of the thermal ramping experiment, heat-induced aggregation only began when the protein had become significantly unfolded. The quadruple mutant was stabilized in such a way that greater unfolding could occur before aggregation was observed. This could result from increased colloidal stabilization, normally associated with increased net charge or decreased surface hydrophobicity. However, the I365L mutation, which induced the observed effect was not expected to alter either property. Alternatively, the increase in *T*_agg_ above *T*_m_ could result from the selective stabilization of a region of structure required to unfold for aggregation to occur, or otherwise from a decrease in the inherent propensity of a local sequence region to form stable intermolecular interactions within aggregates.

The fraction of protein unfolded at 60 °C (*f*_60_) was determined for the WT and mutant TKs, to evaluate the extent to which global protein unfolding influenced the inactivation rates at 60 °C. Surprisingly, *f*_60_ did not show any clear correlation with the *T*_m_ values (Fig. 2*D*). For example, I365L/G506A had the highest *f*_60_ of 0.24, around 0.1 higher than that of WT, and H192P/A282P/G506A had an *f*_60_ of 0.04, around 0.1 lower than that of WT, whereas both variants had higher *T*_m_ and *T*_agg_ values than WT. The lack of correlation between *T*_m_ and *f*_60_ indicated that the cooperativity of unfolding was variable across the mutants, as reflected in their ∆*S*_vh_ values (Table 1). We examined the linear correlations between *T*_m_ and ∆*S*_vh_ value, which indicated that variants with improved *T*_m_ generally had lower ∆*S*_vh_ values, and so apparently lower unfolding cooperativity (*SI Appendix*, Fig. S2). During the thermal unfolding process, the holo-TK homodimer undergoes unfolding of all three domains in each monomer, cofactor release, and also dimer dissociation, apparently at the same time (46). The decreased unfolding cooperativity for variants with increased *T*_m_ could indicate the decoupling of at least one of these events from the rest, due to selective stabilization of one structural element.

### Correlation Between Kinetic Stability and Thermodynamic Stability.

The heat-induced kinetic inactivation of TK proceeds through a second-order reaction, which implies an interaction between at least two molecules, consistent with the observation of aggregation as the end product. Aggregation could proceed through a number of potential mechanisms, including the following: (*i*) molecular reorganization after interaction of native states; (*ii*) partial local unfolding of native states before interaction; or (*iii*) global unfolding before interaction. Thermodynamic stability, as measured by *T*_m_, and more specifically by *f*_60_, can reveal the extent to which global unfolding is important in inactivation by aggregation (47). The deactivation rate constants at 60 °C, expressed as ln(*k*_d_), are shown in Table 1. *T*_m_ gave a good linear correlation to the kinetics of inactivation, with an *R*^2^ value of 0.93 (*SI Appendix*, Fig. S3*A*), indicating a clear link between kinetic and thermodynamic stability. By contrast, the correlation between *f*_60_ and ln(*k*_d_) was poor (*SI Appendix*, Fig. S3*B*), indicating that global unfolding was not the only factor to influence inactivation by aggregation, and likely also involved the decoupling of local unfolding events that manifested as changes in ∆*S*_vh_. In particular, the inactivation rate at 60 °C for H192P/A282P/I365L and H192P/A282P/I365L/G506A decreased significantly, while their fraction unfolded remained at 11–13%, similar to those of H192P/A282P and WT. These two variants had the lowest ∆*S*_vh_ values and also the highest *T*_m_ values of those tested kinetically (Table 1), suggesting that the I365L mutation in particular led to selective stabilization of at least one structural feature that also had a particularly strong stabilizing influence on the inactivation rate.

### Analysis of Epistatic Interactions Between Mutations.

**Fig. 3.**
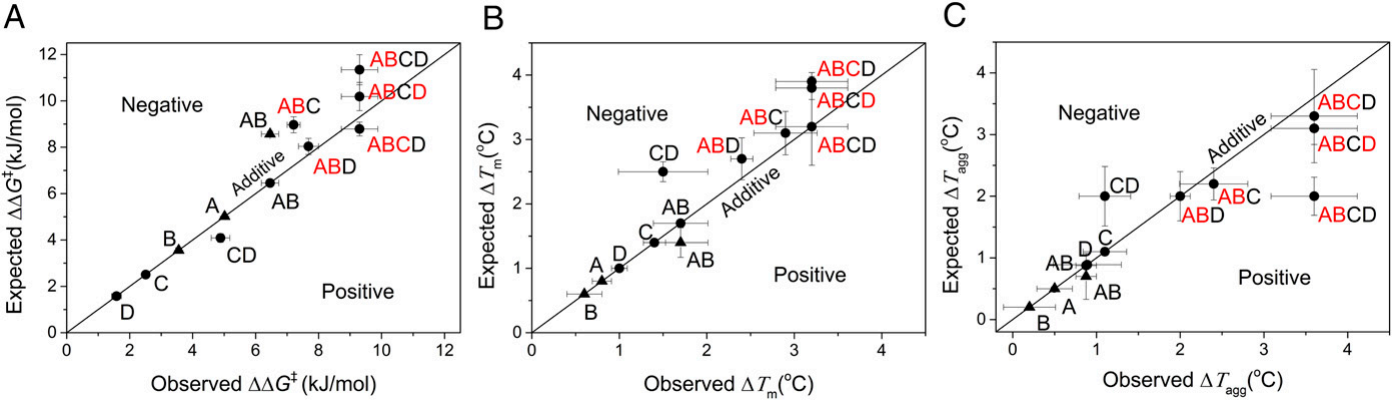
Analysis of epistatic interactions among all of the point mutations based on kinetic and thermodynamic stability. Quantitative analysis of epistatic interactions among the mutations with the ΔΔ*G*^‡^ as the fitness *A*, with the Δ*T*_m_ as the fitness *B*, and with the Δ*T*_agg_ as the fitness *C*. *y* = *x* diagonal reflects the additive effect between two mutations. ABCD could be formed from combinations of AB+CD, C+ABD, D+ABC, and so each combination was indicated by red for the starting variant and black for the new mutation. All of the mutants were represented by letters, and the two evolutionary phases starting from WT, with phase 1 (▲), A—H192P, B—A282P, AB—H192P/A282P; and phase 2 (●), C—Il365L, D—G506A, AB—H192P/A282P, CD—I365L/G506A, ABC—I365L/H192P/A282P, ABD—G506A/H192P/A282P, and ABCD—I365L/G506A/H192P/A282P.

Epistatic interactions between mutations were evaluated for kinetic deactivation (∆∆*G*^‡^) based on the activities retained after heat treatment at 60 °C for 1 h, and also for changes in thermodynamic stabilities, Δ*T*_m_ and Δ*T*_agg_. Fitness landscapes containing the two mutagenic pathways from WT to H192P/A282P (AB), and the six further mutagenic pathways that formed the quadruple-mutant H192P/A282P/I365L/G506A (ABCD), were constructed for all three properties. All pathways were favorable in all three properties, with no local minima due to sign epistasis or reciprocal sign epistasis (*SI Appendix*, Fig. S4). The epistatic interactions between mutations were quantified using Eq. **6** to determine any positive, negative (partially additive), sign, or reciprocal sign epistasis, as shown for ΔΔ*G*^‡^, Δ*T*_m_, and Δ*T*_agg_ in Fig. 3 *A–C*, respectively. H192P (A) and A282P (B) were located on the surface of the PP-domain (2–322 aa) but were 33 Å apart (*SI Appendix*, Fig. S5). Despite this long distance, they showed a strong negative epistasis for ΔΔ*G*^‡^, a moderately positive epistasis for Δ*T*_m_, and an additive effect for Δ*T*_agg_. The two single variants, I365L (C) and G506A (D), were located in the Pyr domain (323–539 aa). The ∆∆*G*^‡^ of the double mutant, CD (4.88 kJ⋅mol^−1^), was higher than the 4.09 kJ⋅mol^−1^ expected from additivity between I365L (C) and G506A (D), indicating a moderately positive epistasis (Fig. 3*A*). The combination of the PP-domain mutations in H192P/A282P (AB), with core mutant (C) to form variant ABC, gave an observed ∆∆*G*^‡^ of 7.21 kJ⋅mol^−1^, with 8.97 kJ⋅mol^−1^ expected from additive effects, indicating negative epistasis between AB and C. Variant ABD gave the ∆∆*G*^‡^ expected from the additive effects of G506A (D) and H192P/A282P (AB). The final variant ABCD was formed via three combinations, ABC+D, CD+AB, and ABD+C. The observed ∆∆*G*^‡^ for ABCD was 9.30 kJ⋅mol^−1^, which was as expected for ABC+D (9.01 ± 0.3 kJ⋅mol^−1^), but lower than the expected values for CD+AB (11.34 kJ⋅mol^−1^) and ABD+C (10.19 kJ⋅mol^−1^), consistent with the negative epistasis found above for AB+C (Fig. 3*A*).

Improvements in *T*_m_ and *T*_agg_ upon combination of I365L (C) and G506A (D) were both negatively epistatic, in contrast to the moderately positive epistasis observed for ΔΔ*G*^‡^ in Fig. 3*A*. The different types of epistasis observed for kinetic (ΔΔ*G*^‡^) and thermodynamic (*T*_m_ and *T*_agg_) stabilities of C+D fits with the observation that kinetic and thermodynamic stabilities were correlated, but that global unfolding was not the only factor to influence kinetic inactivation. I365L and G506A were only 12 Å apart within the same hydrophobic core of the Pyr domain (*SI Appendix*, Fig. S5) and packed onto opposite faces of the indole ring of W503 (*SI Appendix*, Fig. S6). This indirect structural interaction could readily mediate their negative epistasis in Δ*T*_m_ and Δ*T*_agg_, and also their positive epistasis in ΔΔ*G*^‡^. The combination of D+AB showed additive effects for Δ*T*_m_ and Δ*T*_agg_, consistent with ΔΔ*G*^‡^ (Fig. 3). This was expected given that the two mutations in H192P/A282P (AB) were each located on the surface of the PP domain, and nearly 50 Å away from the single mutation G506A (D) in the hydrophobic core of the Pyr domain (*SI Appendix*, Fig. S5). The combination of C+AB showed additive effects for Δ*T*_m_ and Δ*T*_agg_, again in contrast to ΔΔ*G*^‡^, which was negatively epistatic.

The three possible final combination steps leading to ABCD showed distinctly different epistatic effects for ΔΔ*G*^‡^, Δ*T*_m_, and Δ*T*_agg._ As described above, for ΔΔ*G*^‡^, the combination of I365L (C) and H192P/A282P (AB) in any context (C+AB, CD+AB, C+ABD) was negatively epistatic. By contrast, for Δ*T*_m_, negative epistasis was observed, but now for the combination of I365L (C) with G506A (D) in any context (C+D, C+ABD, ABC+D). Finally, for Δ*T*_agg_, while C+D was negatively epistatic, and C+AB was additive, the combination of C and AB at the final step was positively epistatic, particularly for CD+AB. For the pathway to the quadruple mutant, ABCD, all epistatic effects involved I365L (C) and either AB or D, but never occurred specifically between AB and D. As discussed above, the I365L mutation, in the presence of H192P/A282P, also appeared to have a particularly strong stabilizing influence on the inactivation rate, and this contributed to the epistatic effect found between AB+C, AB+CD, and ABD+C. For example, the selective stabilization by I365L of an aggregation-prone motif in the quadruple mutant (ABCD) could lead to the positive epistasis in *T*_agg_, while remaining additive for *T*_m_.

### Cross-Correlations Between Dynamics of Local Regions Mediate Epistatic Interactions.

**Fig. 4.**
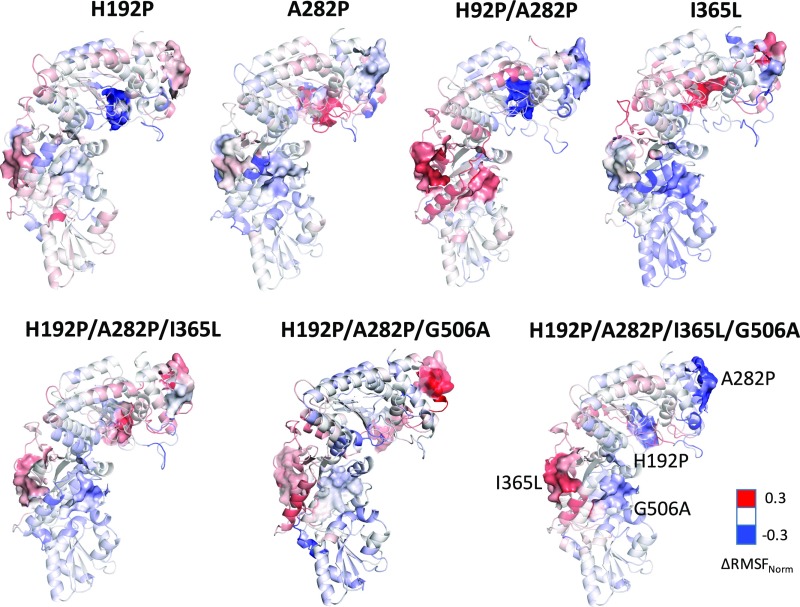
TK variants structures colored by normalized RMSF change with WT. TK variants structures and RMSF values were achieved from the average of last 10-ns MD simulation trajectory at 370 K. The residues within 5 Å around mutation sites were shown with surface. The mutation sites were only labeled on the structure of H192P/A282P/I365L/G506A.

To understand how the mutations interacted to produce both short- and long-range epistasis, we investigated and compared the flexibilities of the WT, H192P, A282P, H192P/A282P, I365L, H192P/A282P/G506A, H192P/A282P/I365L, and the quadruple mutant, H192P/A282P/I365L/G506A, using MD simulations at 370 K. Root-mean-square fluctuation (RMSF) values for each residue indicated a complex interdependence between the dynamics around each mutation, whereby mutations changed their local dynamics, but also sometimes altered the dynamics of certain other, often distant regions (Fig. 4). Previously for the WT TK at 370 K, the PP-binding domain and the C-terminal domain were found to be more flexible by RMSF than the Pyr-binding domain (42). Compared with WT, H192P/A282P had lower flexibility in the PP domain, indicating local stabilization by H192P and A282P mutations (Fig. 4), but increased flexibility in the Pyr domain where residue I365 was located. The introduction of I365L into H192P/A282P led to decreased flexibility around the I365L mutation as expected, but also decreased flexibility within the neighboring C-terminal domain, and conversely increased the flexibility around H192P and A282P. Assuming that increased flexibility at high temperature is correlated with low stability, then this complex interaction between the flexibility of each mutated region would explain the epistasis observed between even distant mutations, such as between I365L and H192P/A282P.

### Dynamics Cross-Correlations Were Consistent Across Variants.

**Fig. 5.**
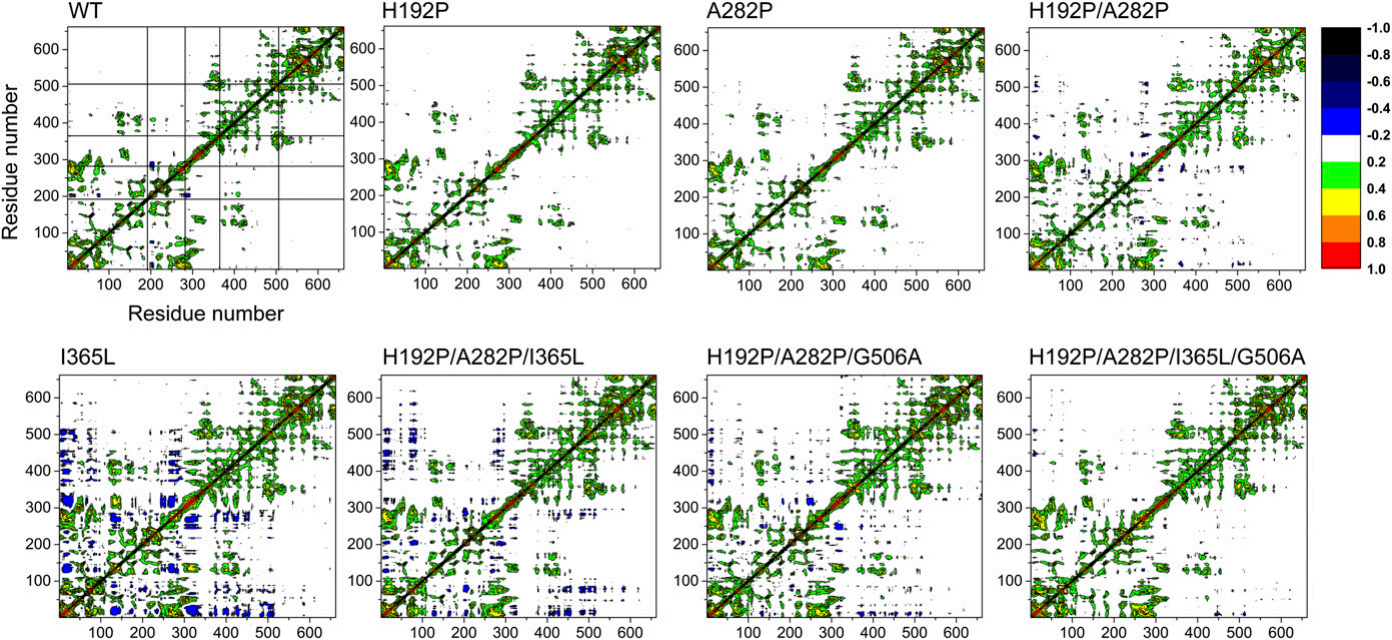
Dynamics cross-correlation map for the C^α^ atom pairs within monomers of TK WT and stable variants. Correlation coefficient (C_ij_) was shown as different colors. C_ij_ with values from 0 to 1 represents positive correlations, whereas C_ij_ with values from −1 to 0 represents negative correlations. Mutated sites are marked for WT with horizontal and vertical lines.

Networks of interactions have been hypothesized to underpin long-range epistasis between mutations, while protein dynamics are known to mediate long-range allostery (25, 27, 29, 30). We therefore computed dynamics cross-correlation matrices (DCCMs) for the WT and variants. Pairwise cross-correlation coefficients (C_ij_) indicate the extent to which the fluctuation of an atom is correlated (or anticorrelated) with one other atom, and dynamics cross-correlation maps show the correlation coefficients (C_ij_) between all C_α_ atom pairs. Most cross-correlations were between structural neighbors (Fig. 5 and *SI Appendix*, Fig. S7), in agreement with the previous observation that cross-correlations decreased with distance (48). Cross-correlations were weak between atoms of the two different monomers, but strong cross-correlations were observed between certain regions within the same monomer. Therefore, we averaged the coefficients from the two chains and investigated the dynamics correlations within the same monomer (Fig. 5). The dynamics of most regions of structure were not correlated to any other region, as seen from significant areas of white space in the DCCM maps. Therefore, any pairwise correlations between distant regions represented an unusual coupling. The locations for correlated dynamics were largely consistent between the WT and variants, such that the mutations did not usually create or remove correlations, although some variants had more anticorrelated zones than the WT (Fig. 5). This indicated that while the mutations modified the RMSF in local and correlated regions, they did not cause any significant disruption to structure or in the networks of interactions linking the mutated regions.

**Fig. 6.**
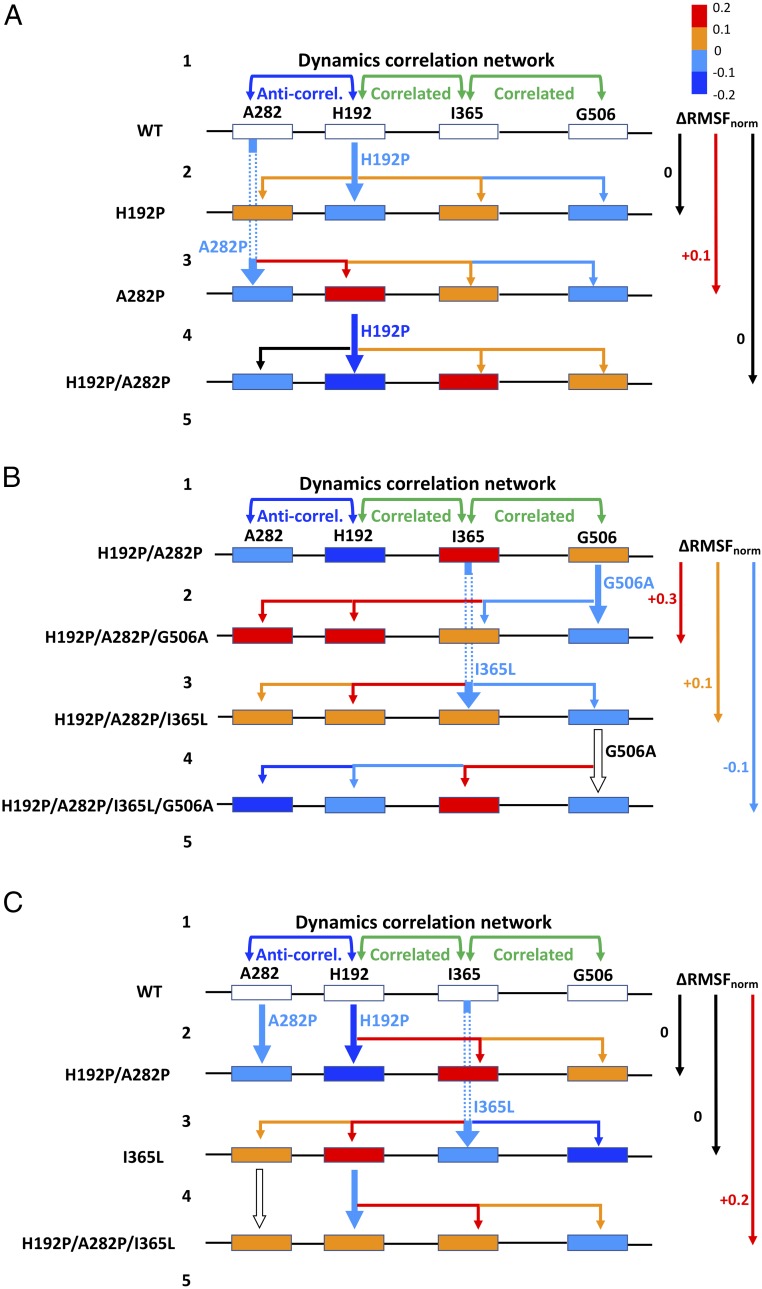
Correlated dynamics link flexibility changes around the four mutations and mediate epistasis between them. Box colors indicate ∆RMSF_norm_ relative to WT at each mutation site, averaged from 11 sequential residues centered on the mutation site. Thick arrows denote mutations. Thin arrows denote impacts on other sites via correlated dynamics. Arrows are colored by ∆RMSF_norm_ upon mutation. *A*–*C* represent single pairwise interactions, with the cause of their epistasis explained in steps 1–5 starting with a common step 1: 1, dynamics correlation network links flexibility of four mutational sites, then (*A*) H192P and A282P. 2, H192P is locally stabilizing. Correlated dynamics destabilizes A282 and I365, and stabilizes G506. 3, A282P is locally stabilizing. Correlated dynamics destabilizes H192 and I365, and stabilizes G506. 4, H192P is locally stabilizing. Correlated dynamics destabilizes at I365 and G506. 5, Negative epistasis in ∆RMSF between H192P and A282P; (*B*) I365L and G506A, within H192P/A282P. 2, G506A is locally stabilizing. Correlated dynamics stabilizes I365 and destabilizes H192P/A282P. 3, I365L is locally stabilizing. Correlated dynamics stabilizes G506 and destabilizes H192P/A282P. 4, G506A now has no impact. Correlated dynamics destabilizes I365L and stabilizes H192P/A282P. 5, Reciprocal sign epistasis in ∆RMSF between G506A and I365L; (*C*) I365L and H192P/A282P. 2, H192P/A282P are locally stabilizing. Correlated dynamics destabilizes I365 and G506. 3, I365L is locally stabilizing. Correlated dynamics stabilizes G506 and destabilizes H192P/A282P. 4, H192P is locally stabilizing. Correlated dynamics destabilizes I365L and G506. 5, Positive epistasis (destabilizing direction) in ∆RMSF between H192P/A282P and I365L.

Pairs of regions with significantly correlated dynamics included between 325–375 and 480–530 aa, between 100–230 and 360–425 aa, and between 0–100 and 240–320 aa. A strong anticorrelation (indicating correlated movement but in opposite directions) was also found between 270–300 and 190–210 aa. Apart from at 0–100 aa, these regions coincided with the four mutation sites, and so the cross-correlated dynamics linked several pairs of mutations (Fig. 6). Specifically, correlation between 325–375 and 480–530 aa linked I365L and G506A, while that between 100–230 and 360–425 aa linked H192P and I365L. The anticorrelation between 270–300 and 190–210 aa linked H192P and A282P. These observations suggest that, while each mutation could modify the local dynamics (RMSF), they could also then alter the dynamics of those regions that were correlated with it. As a result, this would alter the stabilizing impact of a second mutation within that correlated region, if its dynamics were already changed by the presence of the first mutation. This is the basis by which the correlated dynamics between two regions could mediate epistatic interactions between mutations designed to decrease their local flexibilities based upon the WT structure.

The mutations tended to cause significant changes in RMSF (ΔRMSF) only within regions that were strongly correlated to the mutation site via dynamics. A plot of C_ij_ against the ΔRMSF in all residues resulting from each mutation showed that (*i*) most residues do not have dynamics that are strongly correlated with the mutation sites, (*ii*) significant ΔRMSF (>0.2 or less than −0.2) mostly occurs in regions with a strong dynamics correlation coefficient (>0.2) with the mutation site, and (*iii*) the absolute strength and direction of ΔRMSF were not predictable from the pairwise correlation coefficients for the dynamics (*SI Appendix*, Fig. S8). This demonstrates that the correlated dynamics between two regions manifest via specific networks of interactions that induce changes in RMSF within the paired second region, upon mutation within the first region. The link between dynamics correlation and epistasis for several pairs of mutations is described in detail below. By way of example, we examined the impact of each mutation on the local RMSF at only the four mutation sites. This articulates how coupled changes in local RMSF can mediate epistasis between the mutations. Although changes in flexibility in these local regions are expected to contribute to changes in Δ*T*_m_, Δ*T*_agg_, and ΔΔ*G*^‡^, these stability parameters would also depend on the RMSF from other regions of the protein, in a complex manner. Also, the underlying epistasis on local RMSF may manifest in different ways for Δ*T*_m_, Δ*T*_agg_, and ΔΔ*G*^‡^. Therefore, we did not attempt to determine any precise relationship between these and RMSF.

### Dynamics Correlation Between H192P (A) and A282P (B).

The dynamics around H192P were strongly anticorrelated with those around A282P in the WT (Fig. 5). Anticorrelation simply means that atomic motions are correlated such that they move in phase, but in opposite directions (47). The relationship between dynamics correlation and epistasis for the local RMSF at the four mutation sites is outlined schematically in Fig. 6*A*, for the interaction between H192P and A282P. The observed anticorrelation between the local regions around His192 and Ala282 is consistent with the observation that the H192P mutation decreased its local flexibility as anticipated and yet also increased that around A282P, even though it was 33 Å away (Fig. 4 and *SI Appendix*, Fig. S5). By contrast, the A282P mutation decreased its local flexibility and yet also increased that around H192P (Fig. 4). Clearly, this would alter the ability of the H192P or A282P to impart the same stabilizing effects locally within WT, A282P, or H192P, and explains the negative epistasis on ΔΔ*G*^‡^ between H192P and A282P. In both H192P and A282P, the RMSF increased around Ile365 and decreased around Gly506 (Fig. 4), even though the dynamics of His192 were correlated only to those of Ala282 and Ile365, but not to those around Gly506 (Fig. 5). When H192P/A282P was generated from WT, the regions around both Ile365 and Gly506 showed increased flexibility (Fig. 4). As depicted in Fig. 6, the correlations formed a linear network from Ala282 to His192, then on to I365, and finally to G506. Ile365 therefore connected H192P to Gly506 indirectly, consistent with its position in the monomer structure between these two sites. For the total aggregated impact in the four local regions around the mutations (Fig. 6*A*), there was a negative epistasis between H192P and A282P on ΔRMSF.

### Dynamics Correlation Between I365L (C) and G506A (D).

A strong cross-correlation was found between the regions containing Ile365 and Gly506, with a correlation coefficient higher than 0.3 for the WT and all variants (Fig. 5). They are located 12 Å apart in the structure on opposite edges of the indole ring of residue W503 (*SI Appendix*, Figs. S5 and S6). This short network of interactions would readily mediate the correlated dynamics and the epistasis observed between I365L and G506A for all measures of stability, except the *T*_m_ within H192P/A282P. The relationship between dynamics correlation and epistasis for local RMSF is outlined schematically in Fig. 6*B* for the interaction between I365L and G506A within H192P/A282P by way of example. The G506A mutation was locally stabilizing in H192P/A282P, with decreased RMSF in the local region. This led to stabilization at Ile365, then destabilization of H192P and A282P, through the chain of pairwise correlated dynamics between Gly506 and Ile365, Ile365 and H192P, and finally H192P and A282P. Thus, G506A led to a net increase in the RMSF (approximately +0.3 on a normalized scale) aggregated over the four mutation sites (Fig. 6*B*). When the I365L mutation was introduced into H192P/A282P, the RMSF around I365L decreased (Figs. 4 and 6*B*). Correlated dynamics between Ile365 and Gly506 resulted in decreased RMSF around Gly506. Simultaneously, the correlated dynamics between Ile365 and His192 increased the RMSF around H192P, which in turn increased the RMSF at A282P through their anticorrelation. Overall, I365L led to a net increase in the RMSF of approximately +0.1 across the four mutation sites. Both of the single mutations above decreased their own local RMSF but also altered that of other regions coupled by their dynamics. This changes the local baseline RMSF into which the second mutation is made, and therefore leads to epistasis. For example, when G506A was introduced into H192P/A282P/I365L, Gly506 had already been partially stabilized by I365L through their correlated dynamics, and so G506A had only a small impact on the magnitude of the local RMSF. However, the chain of correlated dynamics resulted in increased RMSF at I365L, and decreased RMSF at H192P/A282P, as a result of the G506A mutation. The total ΔRMSF across only the four sites was approximately −0.1, which therefore revealed reciprocal sign epistasis between G506A and I365L. For comparison, these mutations exhibited additivity for Δ*T*_m_, negative epistasis for ΔΔ*G*, and positive epistasis for Δ*T*_agg_. This reemphasizes that the type of epistasis observed for different properties from a given mutational pair are not necessarily the same.

### Dynamics Correlation Between H192P/A282P (AB) and I365L (C).

Ile365 is located 25 and 46 Å from His192 and Ala282, respectively (*SI Appendix*, Fig. S5). In this case, only the coupling of dynamics through a long network of interactions could be expected to cause the observed negative epistasis for ΔΔ*G* and Δ*T*_m_, and positive epistasis for Δ*T*_agg_, between I365L and H192P/A282P. The relationship between dynamics correlation and epistasis for local RMSF is outlined schematically in Fig. 6*C* for the interaction between I365L and H192P/A282P. As above, H192P/A282P were locally stabilizing but led to increased RMSF at both Ile365 and Gly506 (Fig. 4), via the network of correlated dynamics that linked His192 to Ile365, and Ile365 to Gly506 (Fig. 6*C*). Overall, H192P/A282P gave no net change in the local RMSF across the four mutation sites. The I365L mutation decreased the local RMSF when introduced into WT, which then led to stabilization at Gly506, and destabilization around His192 and A282, through their respective pairwise correlated dynamics to I365L. Thus, I365L also gave no net change in local RMSF across the four mutation sites. By contrast, the introduction of H192P/A282P into I365L decreased the RMSF around H192P but induced an RMSF increase around I365L and then Gly506 due to their correlated dynamics, giving a net RMSF increase of approximately +0.2 across the four mutation sites. Therefore, the interaction between H192P/A282P and I365L resulted in positive epistasis for the local ΔRMSF, as a result of coupling between the dynamics of the four local regions. Specifically, the destabilization of H192 and A282 by the distant I365L mutation could no longer be fully rescued by the H192P and A282P mutations, compared with the degree of stabilization that they achieved within WT.

### Insights for the H192P/A282P/I365L/G506A Variant.

A positive epistatic behavior in *T*_agg_ was found between I365L/G506A and H192P/A282P. In contrast to all other variants, the *T*_agg_ of the quadruple mutant, H192P/A282P/I365L/G506A, was higher than its *T*_m_, implying that more unfolding could occur before aggregation was observed. This indicated the selective stabilization of a region of structure required to unfold before aggregation. The MD simulations at high temperature indicated that a remote fragment D81-K96 was rigidified in the quadruple mutant (*SI Appendix*, Fig. S9). The DCCM analysis revealed a strong correlation between this fragment and the region around the A282P mutation, which provided a long-range mechanism through which the mutation could have stabilized the fragment (Fig. 5). This fragment was close to an aggregation hot spot, predicted by three different algorithms (*SI Appendix*, Fig. S9). Stabilization of this region could therefore decrease the propensity of H192P/A282P/I365L/G506A to aggregate, and explain the unusually high *T*_agg_.

### Concluding Remarks.

In this work, we explored the epistatic interaction between the mutations H192P and A282P, located on the surface of the PP domain, and then between the double-mutant H192P/A282P and two single mutations, I365L and G506A, located distantly in the core region of the Pyr domain. Surprisingly, not all pairwise effects between distant mutations from the surface and core regions of different domains were additive. This study has identified and characterized MD that mediated long-range epistatic interactions between mutations for various measures of protein stability. We found that the protein dynamics between the four mutations sites were correlated via a network of interactions that then mediated the observed long-range epistasis. These effects have the potential to be exploited for developing improved protein-engineering strategies. For example, the strategy of rigidifying flexible sites has been proven to be a powerful method to improve the stability of enzymes. When combining mutations that independently improve protein stability, an absence of epistatic interactions might be preferred as this would lead to predictable increases in performance. As mutations with correlated dynamics could potentially interact with each other, protein-engineering strategies could consider combining only those mutations in regions that have no cross-correlated dynamics to maximize the likelihood of additive improvements. Alternatively, experimental and computational protein-engineering approaches may also benefit from deliberately identifying cross-correlated sites. Targeting mutagenic libraries to two or more of these sites simultaneously has the potential to exploit positive epistasis and to rapidly evolve stability via a small number of residues that form a critical network.

## Methods

### Site-Directed Mutagenesis, Overexpression, and Purification of Enzymes.

Primers were designed using the web-based QuikChange Primer Design Program (https://www.agilent.com/genomics/qcpd). Site-directed mutagenesis of tktA within plasmid pQR791 (46), overexpression, and purification of enzymes were carried out as in ref. 42. Protein concentration was measured using the Bradford method (49) and OD_280_ measurements, independently.

### Temperature Inactivation of Holo-TK.

Thermal inactivation was measured as in ref. 42. *T*_50_^15^, the temperature required to reduce the initial enzyme activity by 50% within 15 min, was measured by placing 100 µL of enzymes at various temperatures, from 22 to 65 °C, for 15 min. *T*_50_^15^ was determined from the inflection point of residual activities vs. temperature using a sigmoidal Boltzmann fit in OriginPro 9.0 (50). The half-life of enzyme activity was measured by placing 100 µL of enzymes at 60 °C. Samples were removed at different times and then cooled to 22 °C. Second-order thermal deactivation was fitted to Eq. **1**, where A_(*t*)_ is the activity at time *t* of the heat treatment, A_0_ is the initial activity before heat treatment, and *k* is the inactivation rate constant. Half-life (*t*_1/2_) at 60 °C was calculated as *t*_1/2_ = 1/(100*k_d_). Retained activity (RA) after time *t* of heating was calculated as A_(*t*)_/A_(0)_:

$$\frac{1}{{\text{A}}_{\left(t\right)}}=\frac{1}{{\text{A}}_{0}}+kt.$$ [1]

### Enzyme Kinetics.

Kinetic parameters were obtained at saturating 50 mM Li-HPA and 4–80 mM glycolaldehyde as in ref. 42.

### Thermal Transition Midpoint (*T*_m_) and Aggregation Onset Temperature (*T*_agg_).

Intrinsic protein fluorescence (266-nm excitation, 280- to 450-nm emission scan) and static light scattering (SLS) at 266 and 473 nm, were measured simultaneously with a UNit (Unchained Laboratories) at every 1 °C for 30–90 °C after 30-s equilibration at each temperature. Microcuvettes were loaded with 9 µL of 0.1 mg/mL samples in triplicate. *T*_agg_ was determined from SLS counts at 266 nm using the instrument software. Fluorescence intensity ratio at 350 nm: 330 nm vs. temperature was fitted to a two-state transition model using Eq. **2** (51, 52) in OriginPro 9.0 (Origin Lab Corporation):

$$I_{T}=\frac{I_{N}+aT+\left(I_{D}+bT\right)\exp\left[\left(\frac{\Delta H_{\mathrm{vh}}}{R}\right)\left(\frac{1}{T_{m}}-\frac{1}{T}\right)\right]}{1+\exp\left[\left(\frac{\Delta H_{\mathrm{vh}}}{R}\right)\left(\frac{1}{T_{m}}-\frac{1}{T}\right)\right]},$$ [2]

where *I*_T_ is the observed signal; *I*_N_ and *I*_D_, the native and denatured baseline intercepts; *a* and *b*, the native and denatured baseline slopes; *T*, the temperature; ∆*H*_vh_, the van’t Hoff enthalpy; *R*, the gas constant (1.987 cal⋅mol^−1^⋅K^−1^); and *T*_m_, the thermal transition midpoint. The van’t Hoff entropy was calculated using Eq. **3**, and the mole-fraction, *f*_T_, of unfolded protein at any temperature *T* was calculated from Eq. **4**:

$$\Delta S_{\mathrm{vh}}=\frac{\Delta H_{\mathrm{vh}}}{T_{m}},$$ [3]

$$f_{T}=\frac{\exp\left[\left(\frac{\Delta H_{\mathrm{vh}}}{R}\right)\left(\frac{1}{T_{m}}-\frac{1}{T}\right)\right]}{1+\exp\left[\left(\frac{\Delta H_{\mathrm{vh}}}{R}\right)\left(\frac{1}{T_{m}}-\frac{1}{T}\right)\right]}.$$ [4]

### Analysis of Epistatic Interactions Between Mutations.

Epistasis was quantified using Eq. **5** (53), where ∆∆*G*(X), ∆∆*G*(Y), and ∆∆*G*(X,Y) are changes in free energy relative to WT for single mutations X and Y, and double mutant XY, respectively. ∆*G*_I_ is the coupling energy for interaction between X and Y:

$$\Delta \Delta G_{\left(\mathrm{X,Y}\right)}=\Delta \Delta G_{\left(X\right)}+\Delta \Delta G_{\left(Y\right)}+\Delta G_{I}.$$ [5]

The change in free energy of inactivation relative to WT was calculated from Eq. **6**, where RA_variant_ and RA_WT_ are the activities retained for the variant and WT after the same heat treatment, and A_0_ = 100%:

$${\Delta \Delta G^{‡}}_{\text{variant}}=RT\text{ln}\frac{\left(1-{\text{RA}}_{\mathrm{WT}}\right){\text{RA}}_{\text{variant}}}{\left(1-{\text{RA}}_{\text{variant}}\right){\text{RA}}_{\mathrm{WT}}}.$$ [6]

Epistatic interactions between mutations X and Y were quantified for *T*_m_ and *T*_agg_ using Eq. **7**, and Δ*T*_(X)_, Δ*T*_(Y)_, and Δ*T*_(X,__Y)_ for single mutants X and Y, and double mutant, respectively. Δ*T*_I_ is the epistatic interaction between X and Y:

$$\Delta T_{\left(X,Y\right)}=\Delta T_{\left(X\right)}+\Delta T_{\left(Y\right)+}\Delta T_{I}.$$ [7]

### MD Simulations.

MD simulations of WT TK (Protein Data Bank ID code 1QGD) and variants constructed with the Pymol Mutagenesis Wizard (Schrödinger) were carried out in triplicate at 370 K using Gromacs, version 5.0, exactly as in ref. 42. RMSFs were calculated using the last 10-ns trajectory for analysis of local flexibility.

### Dynamics Cross-Correlation Map.

DCCMs were computed using Bio3D (54, 55). The last-10 ns trajectory from MD simulation was saved at every 10 ps and converted to a dcd file type with the VMD plugin CatDCD (56), input to Bio3D, and the C^α^ atoms selected for calculating the correlation coefficients. Dynamics correlation matrices were averaged from triplicate trajectories and visualized using OriginPro9.0.

## Supplementary Material

Supplementary File
