# Supplementary material for: Coupled molecular dynamics mediate long- and short-range epistasis between mutations that affect stability and aggregation kinetics
Source: Proc Natl Acad Sci U S A. 2018 Nov 7;115(47):E11043–52. doi: 10.1073/pnas.1810324115 (PMC6255212; doi:10.1073/pnas.1810324115)
Supplement: Supplementary File [file pnas.1810324115.sapp.pdf]

PNAS
www.pnas.org

Supplementary Information for

Coupled molecular dynamics mediate long and short-range epistasis between mutations that affect stability and aggregation kinetics

Haoran Yu, Paul A. Dalby

Department of Biochemical Engineering, University College London, Gordon Street, London, WC1H 0AH, United Kingdom

Correspondence and requests for materials should be addressed to P. A. D.
Email: p.dalby@ucl.ac.uk

This PDF file includes:

Supplementary text
Figs. S1 to S9
Tables S1
References for SI reference citations

www.pnas.org/cgi/doi/10.1073/pnas.1810324115

Methods

Aggregation hotspots prediction The aggregation propensity of TK was predicted using three tools including AGGRESCAN (1), PASTA (2) and TANGO (3). The FASTA format sequence from crystal structure (PDB ID 1QGD) was used as the input for the AGGRESCAN and PASTA. As for the TANGO, in addition to the peptide sequence, the input parameters also included the pH of 7.0, temperature of 298.15 K, ionic strength of 0.05 and no protection at terminals. In its output, the segment with an aggregation tendency above 5% over 5-6 residues was considered as the potential aggregation hotspots.

Distance measurement between mutations. The PyMol molecular graphics system (Schrödinger, USA) was used calculate the distances between α-carbon atoms of mutant residues with the structure of TK (PDB ID 1QGD) as the input.

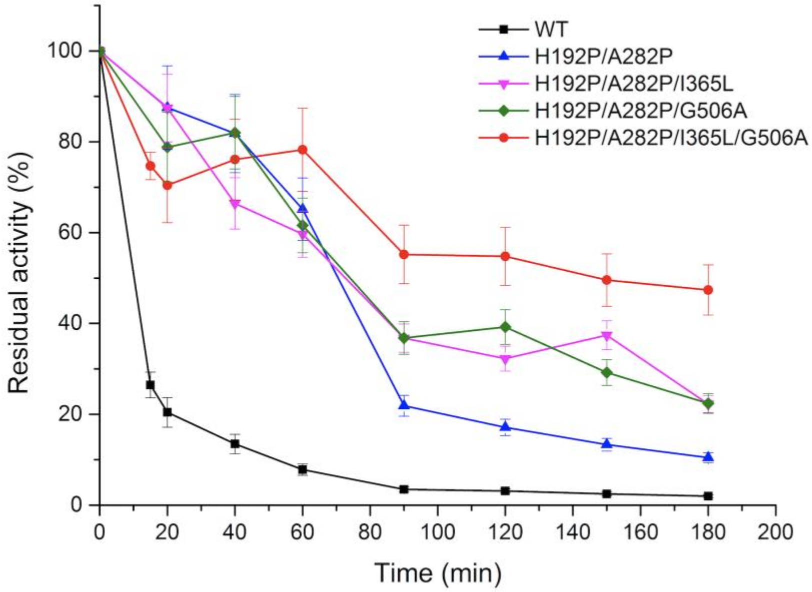

Fig. S1. Inactivation profiles of *E. coli* TK and its variants at 60 ºC. Enzymes in 100 µL solution (2.4 mM TPP, 9 mM $MgCl_2$ and 50 mM Tris-HCl, pH 7.0) were incubated at 60 ºC for different time and assayed for retained activity at 22 ºC.

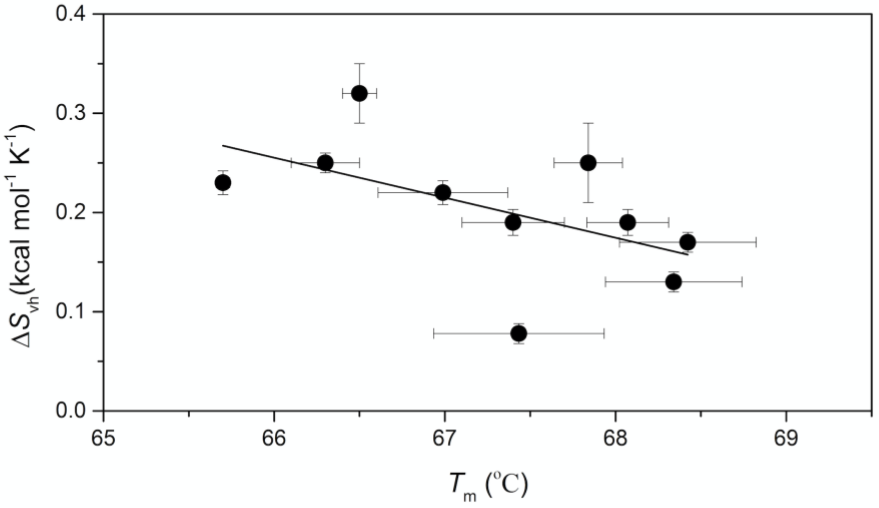

Fig. S2. Van't Hoff entropy of TK variants decreases as the T_m increases. Measurements were derived from intrinsic fluorescence at 0.1 mg/mL enzyme in 50 mM Tris-HCl, 2.4 mM ThDP, 9 mM $MgCl_2$, pH 7.0, for the variants listed in the Table 1.

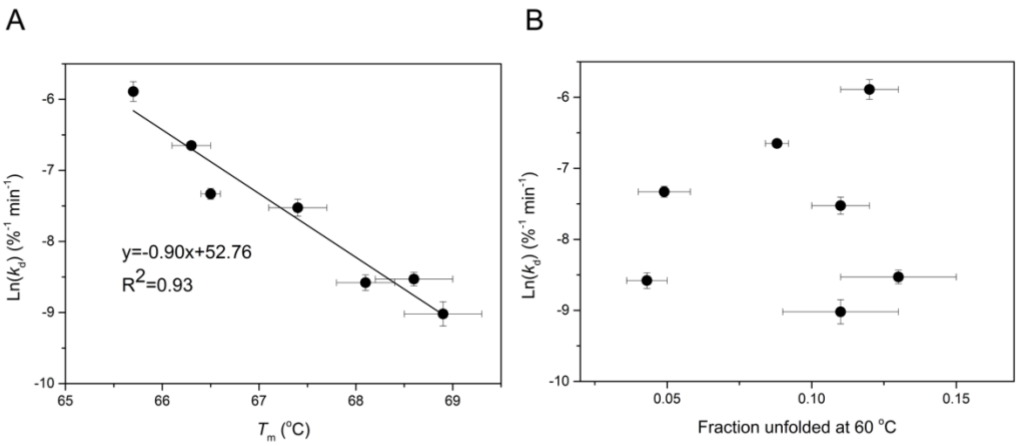

Fig. S3. Correlation between T_m or f_{60} and initial rate of deactivation for TK variants. A, correlation between T_m and ln(k_d). B, correlation between f_{60} and ln(k_d). (●) variants include WT, H192P, A282P, H192P/A282P, H192P/A282P/I365L, H192P/A282P/G506A, H192P/A282P/I365L/G506A.

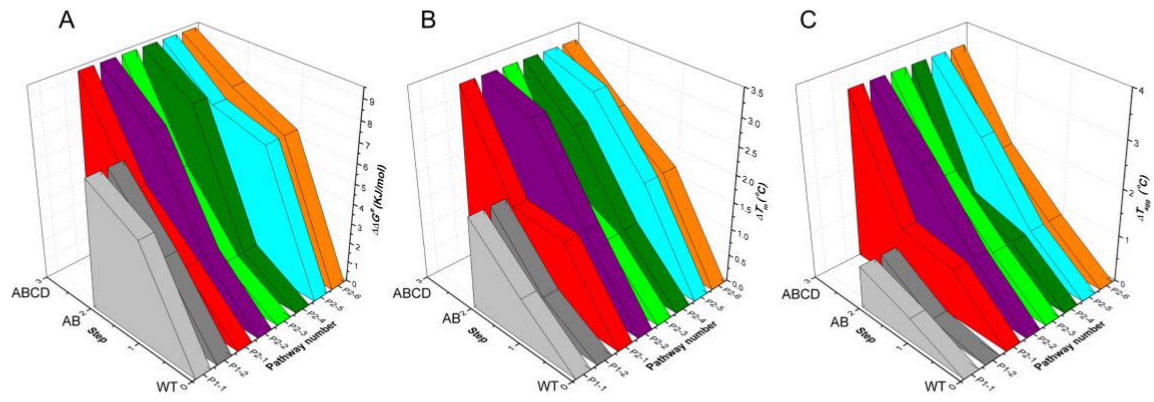

Fig. S4. Fitness-pathway landscape featuring 8 pathways from WT to the thermostable mutant I365L/G506A/H192P/A282P with the $\Delta\Delta G^{\ddagger}$ as the fitness A, with the ΔT_m as the fitness B, with the ΔT_{agg} as the fitness C. P1-1 A-AB (grey); P1-2 B-AB (dark grey); P2-1 C-CD-ABCD (red); P2-2 C-ABC-ABCD (purple); P2-3 D-CD-ABCD (green); P2-4 D-ABD-ABCD (dark green); P2-5 AB-ABC-ABCD (cyan); P2-6 AB-ABD-ABCD (orange). All the mutants were represented by letters, and the two evolutionary phases starting from WT, with phase 1: A-H192P, B- A282P, AB-H192P/A282P; and phase 2: C-Il365L, D-G506A, AB-H192P/A282P, CD-I365L/G506A, ABC-I365L/H192P/A282P, ABD-G506A/H192P/A282P, ABCD-I365L/G506A/H192P/A282P.

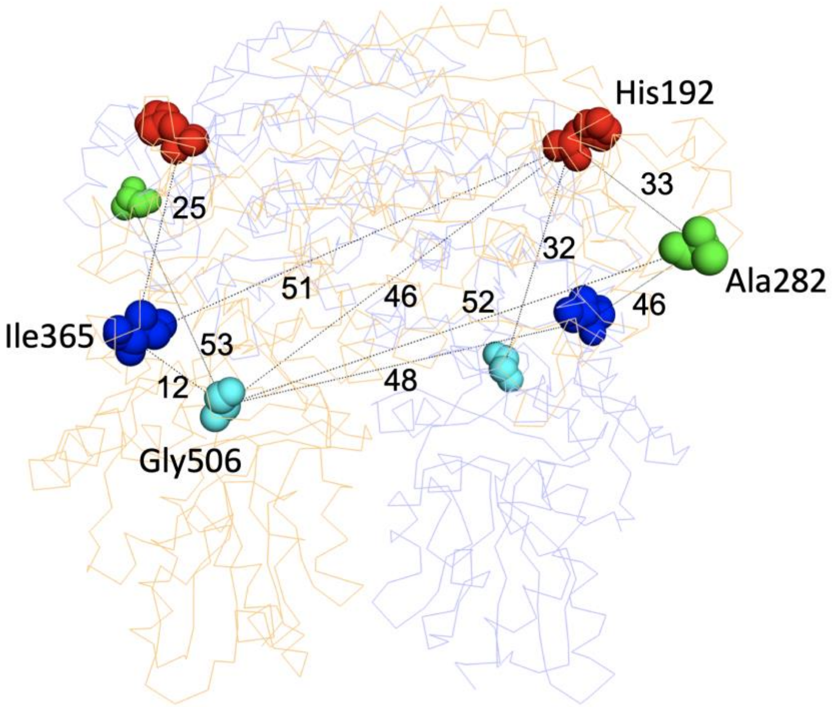

Fig. S5. Distances between mutant residues shown on the x-ray structure of *E. coli* TK. Image was generated in *PyMOL* and only the residues in one monomer were labelled. The distances in angstrom were measured between atom C^{α} of residues. The values were rounded to the integers. (His192, red; Ala282, green; Ile365, blue; Gly506, cyan).

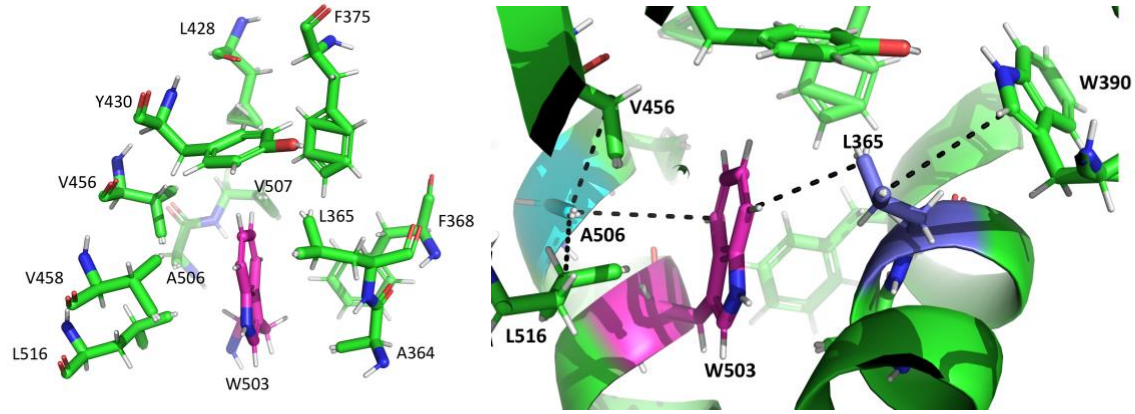

Fig. S6. The hydrophobic core around W503 in the variant of H192P/A282P/I365L/G506A. Dash lines indicate hydrophobic interactions.

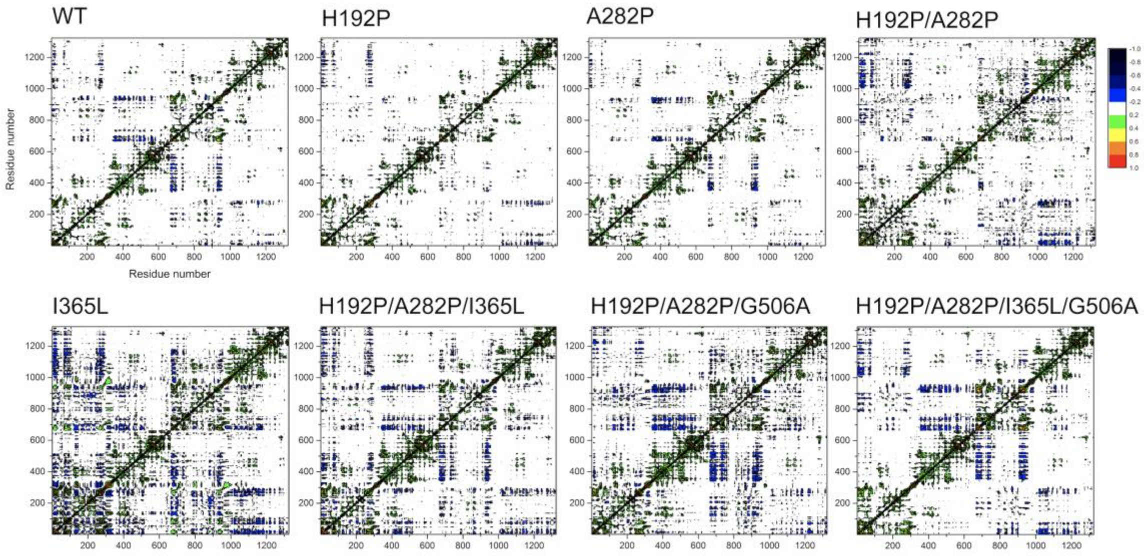

Fig. S7. Dynamics cross-correlation map for the C_α atom pairs of full homodimers for TK WT and stable variants. Chain A, residue number 1-663; Chain B, residue number 664-1326. Correlation coefficient (C_{ij}) was shown as different colours. C_{ij} with values from 0 to 1 represents positive correlations, whereas Cij with values from -1 to 0 represents negative correlations.

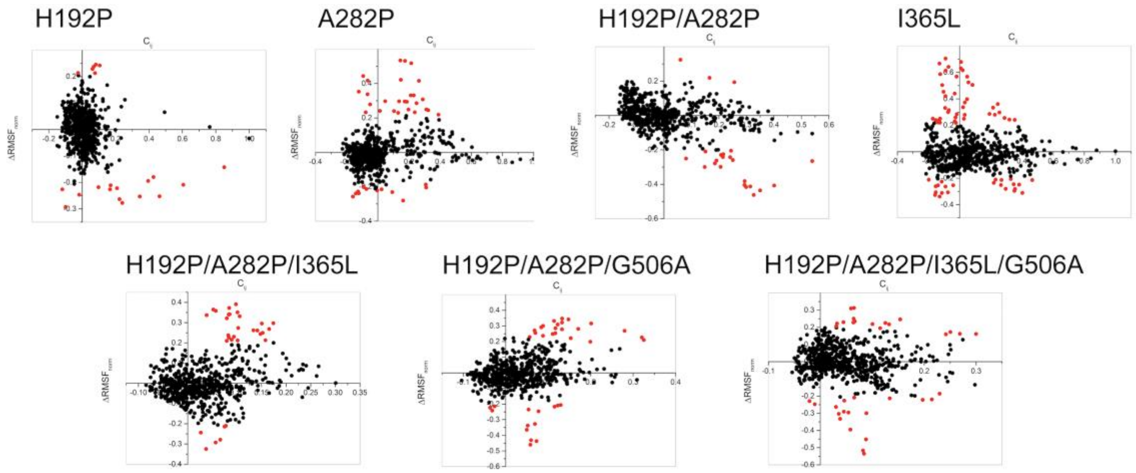

Fig. S8. Comparison of the ΔRMSF at each residue, and the dynamics correlation coefficient (C_{ij}) between that residue and the mutated sites within each TK variant. The residues showing significant ΔRMSF were colored red. For the recombined variants, the C_{ij} was the average from all single mutations.

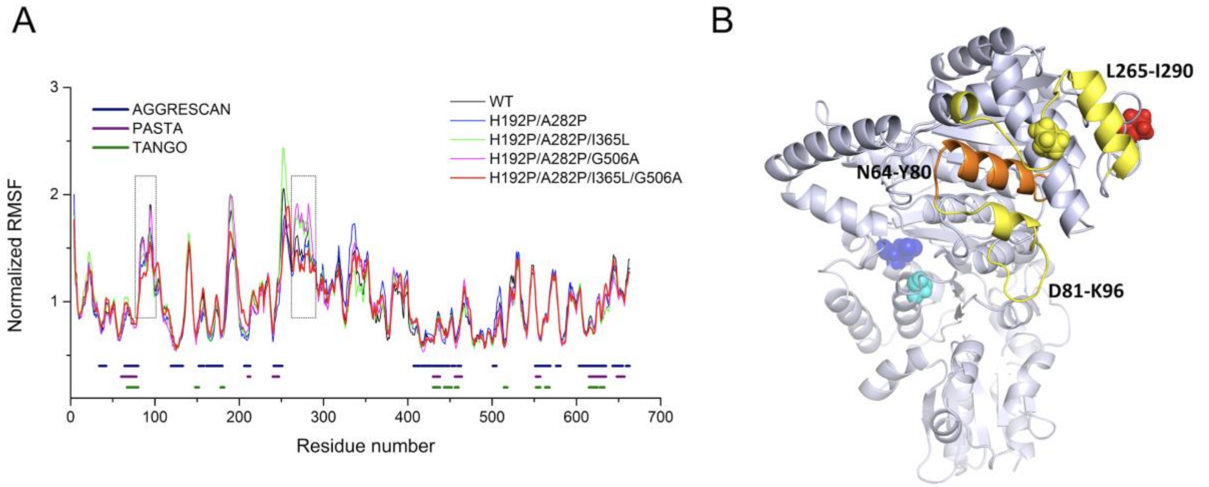

Fig. S9. Unusually high T_{agg} value of the mutant I365L/G506A/H192P/A282P. A, the aggregation hotspots predicted by AGGRESCAN, PASTA and TANGO were shown. B, the local structure around the fragment D81-K96 in the quadruple variant. Four mutations H192P, A282P, I365L, G506A were shown as spheres and coloured by red, yellow, blue and cyan, respectively.

Table S1. Kinetics of wild-type and mutant TKs

	K_m (mM)	k_{cat} (s^{-1})	k_{cat}/K_m (s^{-1} M^{-1})
WT	20.3(2.2)	62.3(2.4)	3069
H192P/A282P/I365L	19.5(2.1)	68.9(2.6)	3533
H192P/A282P/G506A	20.2(1.5)	54.3(1.4)	2688
H192P/A282P/I365L/G506A	22.7(2.2)	58.3(2.1)	2568

References

1. Conchillo-Sole O, *et al.* (2007) AGGRESCAN: a server for the prediction and evaluation of "hot spots" of aggregation in polypeptides. *BMC Bioinformatics* 8:65.
2. Trovato A, Seno F, & Tosatto SC (2007) The PASTA server for protein aggregation prediction. *Protein Eng Des Sel* 20(10):521-523.
3. Fernandez-Escamilla AM, Rousseau F, Schymkowitz J, & Serrano L (2004) Prediction of sequence-dependent and mutational effects on the aggregation of peptides and proteins. *Nat Biotechnol* 22(10):1302-1306.
